# A Review of Caffeine Adsorption Studies onto Various Types of Adsorbents

**DOI:** 10.1155/2021/9998924

**Published:** 2021-07-19

**Authors:** Javier Andrés Quintero-Jaramillo, Javier Ignacio Carrero-Mantilla, Nancy Rocío Sanabria-González

**Affiliations:** Departamento de Ingeniería Química, Universidad Nacional de Colombia Sede Manizales, Campus La Nubia, km 7 vía al Aeropuerto, AA 127, Manizales, Colombia

## Abstract

A systematic literature review of publications from 2000 to 2020 was carried out to identify research trends on adsorbent materials for the removal of caffeine from aqueous solutions. Publications were retrieved from three databases (Scopus, Web of Science, and Google Scholar). Words “adsorption AND caffeine” were examined into titles, abstracts, and keywords. A brief bibliometric analysis was performed with emphasis on the type of publication and of most cited articles. Materials for the removal of caffeine were classified according to the type of material into three main groups: organic, inorganic, and composites, each of them subdivided into different subgroups consistent with their origin or production. Tables resume for each subgroup of adsorbents the key information: specific surface area, dose, pH, maximum adsorption capacity, and isotherm models for the removal of caffeine. The highest adsorption capacities were achieved by organic adsorbents, specifically those with granular activated carbon (1961.3 mg/g) and grape stalk activated carbon (916.7 mg/g). Phenyl-phosphate-based porous organic polymer (301 mg/g), natural sandy loam sediment (221.2 mg/g), composites of MCM-48 encapsulated graphene oxide (153.8 mg/g), and organically modified clay (143.7 mg/g) showed adsorption capacities lower than those of activated carbons. In some activated carbons, a relation between the specific surface area (SSA) and the maximum adsorption capacity (*Q*_max_) was found.

## 1. Introduction

Emerging contaminants (ECs) include a wide range of chemical compounds, pharmaceuticals, personal care products, surfactants, industrial additives, plasticizers, and pesticides, among other, and their possible consequences for human health and the environment effects in many cases are still unknown [1–4]. The problem with ECs present in water sources is that plants for purification and wastewater treatment are not able to eliminate them completely, so their persistence in the environment is continuous. Caffeine is continuously detected, due to the advancement and development of instrumental analysis methods [5], and it is considered an indicator of anthropogenic contamination due to its common use by peoples [6]. Caffeine is an EC commonly found in drinking water [7–9], groundwater [4], wastewater [10–12], effluents from wastewater treatment plants [12], rivers, lakes, seas [13], and even in the Antarctic waters [14]. Caffeine is a persistent compound, routinely detected even in countries where coffee is not cultivated but consumed. For example, caffeine concentrations of 0.29 *μ*g/L and 564 ng/L were determined in drinking water in California (USA) [9] and China [15], respectively. Also, caffeine concentration values in the 1644–3344 ng/L range have been measured in the Italian river Lambro after receiving the wastewater discharge from the city of Milan [16].

**Figure 1:**
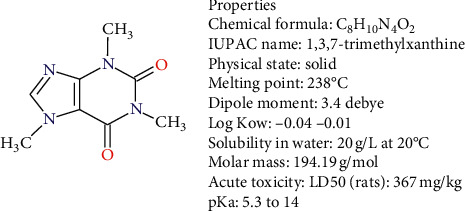
Chemical structure and properties of caffeine [17, 18].

Caffeine is a stimulant of the central nervous system, a chemical compound from the group of methylxanthines (as illustrated in Figure 1), and the mostly consumed psychoactive substance in the world [19]. Caffeine is a naturally occurring alkaloid in approximately 60 plant species, including coffee, tea, and cocoa [20]. It also appears in some analgesic and bronchodilator drugs, and even in shampoos [6, 17, 18].

According to the information in Figure 1, the dipole moment of caffeine is high, increasing with the polarity of the medium that holds it [21]. It means that the positive charge of nitrogen in caffeine electrostatically interacts with any negatively polarized functional group [17, 22]. The pKa and pH affect the chemical behavior of pollutants, and a protonated form of caffeine will be produced when pH < pKa [23, 24]. The pKa and pH affect the chemical behavior of contaminants, so a protonated form of caffeine will be produced when pH < pKa.

Chemical treatments for emerging contaminants in water, as caffeine, include ozonation, photo-Fenton processes, photoelectrolysis, and electrochemical oxidation [15, 17, 25, 26]. However, these technologies tend to consume high energy and do not achieve a complete mineralization [27]. In contrast, adsorption is efficient, inexpensive, versatile, and environmentally friendly. Adsorption is widely used to remove contaminants in water, especially those that are not biodegradable such as heavy metals and ECs, being caffeine one of them [28–32]. Recent proposals for the removal of caffeine include batch and fixed bed adsorption treatments [26, 33]. Powdered activated carbon (PAC) and granular activated carbon (GAC) have been the most widely used adsorbents due to their high specific surface area and chemical surface properties.

Adsorption is a surface phenomenon, where contaminants dissolved in a liquid phase (adsorbate) interact with a porous solid surface (adsorbent). Generally, the adsorbent surface contains functional groups that allow physical or chemical interaction with the adsorbates present in the fluid [34]. In industrial processes, the contaminated fluid passes through a fixed bed where solid adsorbate particles retain the contaminant. When the bed becomes almost saturated, it regenerates inducing desorption of the adsorbate by heating or other methods, so the adsorbent becomes ready for another adsorption cycle [35]. Adsorption is influenced by several factors, including pH, ionic strength, temperature, amount of adsorbent, particle size, contact time, initial solute concentration, specific surface area, and stirring speed [36–38]. The selection of the operating ranges for these factors is essential in the study of the adsorption process [39].

Interest in the use of adsorbents for the removal of caffeine from aqueous solutions has led to the publication of several reviews on topics related to the very same purpose of this paper. Anastopoulos et al. [40] published a review focusing on emerging contaminants such as caffeine, nicotine, and amoxicillin. That review covered the toxic effect of caffeine in humans and animals and described some of the main adsorbents used for its removal. Isothermal and kinetic models for the analysis of caffeine adsorption were also presented, as well as the maximum adsorption capacity and possible adsorption mechanisms. Rigueto et al. [17] prepared a review on methods for removing caffeine from aqueous solutions and real effluents, examining major findings and limits for each process. The research concluded that, despite encouraging application trends, current technologies for caffeine removal have significant limitations, including the complexity of adsorption mechanisms, quantification of contaminants in real effluents, and the low sustainability of the technique. Finally, Bachmann et al. [41] performed a systematic review of the removal of caffeine by adsorption, emphasizing on the evolution of adsorbents used and the kinetic, equilibrium, and thermodynamic studies. The pseudo-second-order (kinetic model) and the Langmuir isotherm models yield the best fit of the experimental data in most studies. On the other hand, our review presents a brief bibliometric analysis of publications of the last two decades, a classification of adsorbents in three groups, including a novel analysis of the process variables (pH and mass of adsorbent), characterization of the adsorbent (SSA), and the maximum adsorption capacity (*Q*_max_).

Current trends in caffeine adsorption point the development of materials with adsorbent properties (affinity for the pollutant and high specific surface area), which are abundant and inexpensive. In this sense, the strengthening of scientific research related to the application of new adsorbent materials for the removal of caffeine, contributes to the implementation of clean and environmentally friendly industrial technologies and processes [32, 40, 42, 43]. This document provides a systematic review of literature about caffeine adsorption, presenting the diversity of adsorbents used in research reported in the last two decades, as well as a brief bibliometric analysis. An analysis of the results of the specific surface area of adsorbents was performed, as well as the variables that affect the adsorption process, specifically pH and adsorbent mass (pH is commonly controlled and evaluated in studies of caffeine adsorption). The results of the maximum adsorption capacities and their adjustments to the model's adsorption isotherms are also presented. This review article will allow researchers to identify the types of adsorbents mostly used for caffeine adsorption in aqueous medium, in addition to adsorption conditions such as adsorbent dose and pH. An analysis of the maximum adsorption capacity (*Q*_max_) and its relationship with the specific surface area (SSA) of each adsorbent is also shown to determine the performance of the different types of materials used for adsorption.

## 2. Materials and Methods

A systematic literature review of publications (research articles, review articles, book, book chapters, and conference papers) from 2000 (January 1) to 2020 (July 29) was carried out to identify the research trends on caffeine adsorption from aqueous solutions, emphasizing the adsorbent materials. During the period 2000–2020, there was an increase in the number of publications on adsorption of different ECs and, although caffeine is mentioned, the first found document that studies caffeine adsorption was published in 2004 [44].

The references were obtained from three search and indexing databases: Scopus, Web of Science, and Google Scholar, which together cover approximately 95% of the publications worldwide. Scopus indexes the largest number of journals (20% more coverage than WoS), but WoS performs a more open search so the filters had to be more specific and personalized [45, 46]. These databases included research articles, review articles, books, book chapters, and conference papers. The search was performed in the title, abstract, and keywords of publications. For search equation “caffeine AND adsorption,” 528 results were found.

The 528 preliminary results were manually filtered to remove repeated articles in the databases, to exclude publications that were not within the scope of the present work, and most importantly, to identify the most relevant publications. Finally, 133 publications were used for the preparation of this review, mainly journal articles on environmental sciences, chemistry, chemical engineering, engineering, and material sciences.

## 3. Results and Discussion

### 3.1. Brief Bibliometric Analysis

**Figure 2:**
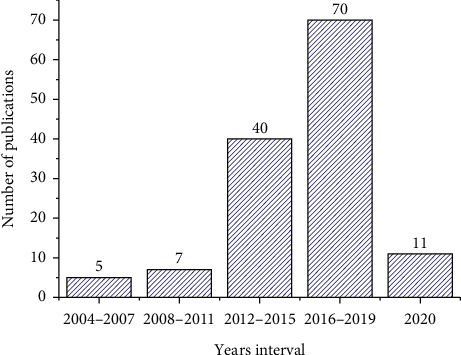
Number of publications related to the removal of caffeine from water using adsorption per quadrennium.

A bibliometric analysis of the 133 publications selected in the search strategy was performed.  Figure 2 shows the number of publications on caffeine adsorption per quadrennium from 2004 to 2020. The period between 2012 and 2019 corresponds to 82.7% of the total number of publications, which suggests that it is a current research topic. For the year 2020 (until July 29) there are 11 publications, a similar value to the number of publications between 2004 and 2011.

The distribution of publications in Table 1 shows that most publications are research articles, concentrated in the areas of are chemistry, environmental science, and chemical engineering, which add up to 56.3% of the total number of publications. Most of the publications (52.8%) come from three countries: China, Spain, and the USA. Besides, 93.8% of all publications are published in English, while 4.9% in Chinese, and the remaining in other languages: Spanish, French, Japanese, German, Portuguese, and Italian. Two-thirds of the publications on caffeine adsorption are concentrated on four journals: Science of the Total Environment (23.1%), Chemosphere (18.7%), Environmental Science (13.2%), and Chemical Engineering Journal (12.1%). Also, a ranking of institutions per number of publications on caffeine adsorption is shown in Table 2.

The ten most cited publications are listed in Table 3, eight of them describe remotion with carbonaceous adsorbents: graphene nanoplatelets, activated carbons, and carbon xerogels. The remaining two publications describe the application of low-cost adsorbents: carbon fibers prepared from pineapple plant leaves, and natural sediments.

**Figure 3:**
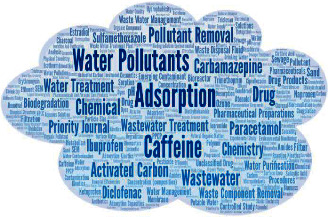
Word cloud view of the most frequently used keywords for caffeine adsorption.

To conclude the bibliometric analysis, the cloud shown in Figure 3 summarizes the appearance of keywords in the publications. The five most used keywords were *adsorption* (102 times), *caffeine* (94 times), *water pollutants* (54 times),* drug *(21 times), and *activated carbon* (19 times). Therefore, this word cloud illustrates the interest in water and wastewater treatment and the use of adsorbents such as activated carbons for the removal of pharmaceutical products (caffeine, ibuprofen, diclofenac, and carbamazepine). The term pH also stands out, indicating that it is one of the most frequently studied variables in caffeine adsorption processes, being a keyword in 20 of the 133 publications. Finally, the appearance of *kinetic* and *isotherm* highlights the interest in understanding the caffeine adsorption mechanism.

### 3.2. Classification of Adsorbents

**Figure 4:**
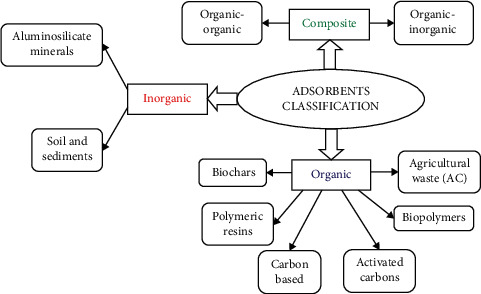
Classification of adsorbents for caffeine adsorption.

All the adsorbents mentioned in the publications follow the common definition, i.e., solid materials with micro- and mesopores that can take a significant part of the material volume [56, 57].  Figure 4 shows the classification of adsorbents in three groups (with their respective subgroups):

  Organic (68.3% of publications): carbon-based, they can be of natural origin such as agricultural residues and biochar or of synthetic origin such as polymers. Some of them are of mixed origin, such as biopolymers obtained from chitosan compounds [58].
  Inorganic (20.8%): mainly minerals, for example, silica, metal oxides, and materials such as mineral clays, sediments, and soils. By origin, they can be synthetic or natural [59].
  Composite (10.9%): hybrids that combine two or more materials, of organic and/or inorganic type [60]. This group of adsorbents have been investigated with promising results for the removal of dyes [61, 62], heavy metals [63, 64], and emerging contaminants [65, 66].

For each subgroup of adsorbents, the following parameters were analyzed: specific surface area (SSA), adsorbent dose, pH, maximum adsorption capacity, and isothermal and kinetic models for caffeine adsorption to which the data were adjusted.

#### 3.2.1. Organic Adsorbents

The summary of characteristics of the subgroups of organic adsorbents and conditions of the adsorption process are shown in Table 4 (activated carbons), Table 5 (carbon-based), Table 6 (agricultural wastes directly as adsorbents or as precursors for activated carbons), Table 7 (biochar), and Table 8 (polymeric resins and biopolymers). Most of the organic adsorbents in this group are activated carbons (Table 4), either powdered (PAC) or granular (GAC). Activated carbons have a high specific surface area, between 578 and 2431 m^2^/g and good surface chemical properties, but they are expensive, and some of them cannot be regenerated [67]. Their adsorption capacity range is very wide, including low (161 ng/g–396 *μ*g/g), medium (4.95–219.2 mg/g), and high (271–1961.26 mg/g) values. The pH described for caffeine adsorption varies between 3 and 9 for this subgroup of adsorbents, with the highest *Q*_max_ values obtained at pH 7.

The carbon-based adsorbents subgroup (Table 5) covers materials that come from carbon but differ from activated carbons, such as xerogels, nanotubes, nanofibers, and graphene. Carbon-based adsorbents have a smaller specific surface area and removal capacity than activated carbons, although they are considered materials with potential application for the removal of caffeine [48, 72, 82–84]. Carbon cloth has both the highest SSA and *Q*_max_ values in this subgroup, 1560 m^2^/g and 369.0 mg/g, respectively [85].

Carbon xerogels and carbon cloth show adsorption capacities of 107.0 and 369.0 mg/g, respectively [52, 85], while carbon nanotubes and commercial column C18 show adsorption capacities in the range of 8.14 to 41.6 mg/g and less specific surface area [72, 82, 83, 86]. Following Table 5, the most used pH for removal of caffeine is close to neutrality, except for carbon xerogel modified with (CH_3_COO)_2_Cu, nanotubes, and carbon nanofibers, where the optimal pH at values of 2 and 3 was found [72, 87].

Agricultural wastes (Table 6) are low-cost natural raw materials, abundantly available. The agricultural waste subgroup includes them used directly as adsorbents and the activated carbons produced from them, which have improved properties, mainly specific surface area (407.66–1099.86 m^2^/g) and adsorption capacity for caffeine (8.7–916.7 mg/g).

Activated carbons are produced from agricultural wastes by controlled pyrolysis combined with chemical treatment, taking advantage of its high carbon content and low percentage of inorganic matter [88–90]. Activated carbons from grape stalk and pine have the highest SSA and adsorption capacity [91, 92], while those obtained from other agricultural wastes such as peach stones, dende coco, and babassu coco have a *Q*_max_ between 186.9 and 270 mg/g, which is anyway higher than those of other carbon-based adsorbents such as nanotubes (41.6 mg/g) and nanofibers (28.3 mg/g) [72].

Regarding pH, acidic pHs in the range 2–5 tend to yield the best results, such as produced as follows: at pH 2, a maximum adsorption capacity of 13 mg/g for the activated carbon obtained from *Elaeis guineensis* (palm oil) [93]; at pH 3, a *Q*_max_ of 212.3 mg/g for biomass-derived activated carbons (dende coco and babassu coco) [94]; at pH 4, a *Q*_max_ of 916.7 mg/g for grape stalk activated carbon [92]; and at pH 5, a *Q*_max_ of 500 mg/g for pine activated carbon with K_2_CO_3_ [91]. For pH values between 6.3 and 7.7, a *Q*_max_ of 30.3 to 270 mg/g has been reported, being the case of activated carbons obtained from residues of *Acacia mangium* wood, pineapple plant leaves, *Eragrostis plana*, and peach stones [51, 54, 95, 96].

Biochar adsorbents (Table 7) are obtained from pyrolysis of animal or vegetable biomass at temperatures between 300 and 700°C with a low oxygen amount [102–104]. The interest in biochar adsorbents has recently surged due to their high specific surface area and adsorption capacity [105, 106]. The adsorption capacities of the biochar adsorbent subgroup (Table 7) are between 6.54 and 40.2 mg/g, well below values of activated carbons but comparable with those of carbon-based adsorbents such as nanoplatelets [48], carbon nanofibers [72], and graphene [84]. The pH for caffeine adsorption in this subgroup of adsorbents is slightly acidic, with values between 3.5 and 5.9.

Polymeric resins, solid or liquid, are usually based on polystyrene, polyacrylamide, and polyvinyl alcohol. Recent improvements in polymerization processes have achieved enough resistance and chemical stability to make them suitable for the removal of contaminants from water [60]. Summary of characteristics of polymeric resins and biopolymers and conditions of the adsorption process are described in Table 8. The *Q*_max_ in Table 8 is not very high (between 256.4 ng/g to 301 mg/g) compared with the *Q*_max_ of activated carbons, but the high specific areas of the polymer resins (450–1000 m^2^/g) suggest that they could be very effective for the adsorption of other contaminants.

Biopolymer adsorbents are polymers obtained from algae, plants, or agro-industrial products, hence the bio prefix. The most common one is chitosan, which is obtained from chitin, an abundant mucopolysaccharide and the support material of crustaceans and insect exoskeletons. It is biocompatible, biodegradable, and nontoxic and, most importantly, has good adsorption properties [122]. Chitosan and other biopolymers have been evaluated with good results for removal of contaminants such as metals and dyes, but their performance for caffeine adsorption is not promising [123–125]. The costs of obtaining biopolymers are very high and show no advantages over other adsorbents investigated for the removal of caffeine, except when used with other materials to form composites [123, 126].

#### 3.2.2. Inorganic Adsorbents

This kind of adsorbents can be classified into two subgroups: aluminosilicate minerals (Table 9) and soil and sediment adsorbents (Table 10); and the most used for the removal of caffeine have been clay minerals (natural and modified), zeolites, soils, and sediments. It is also possible to change the naturally hydrophilic character of smectite clay minerals into organophilic, making them act as adsorbents for organic compounds. The intercalation of surfactant cations (quaternary ammonium salts) into layers of clay minerals not only changes the surface properties but also increases the interlamellar distance (basal spacing) of the layers, thus easing the adsorption of organic molecules [42, 59, 127–131]. Bentonite (a montmorillonite) and saponite are the most frequently used clays as caffeine adsorbents.

Regarding aluminosilicates (Table 9), smectite minerals—such as montmorillonite and saponite and their modifications, organoclays, or thermally treated smectites—show a good caffeine adsorption capacity (99.0–143.7 mg/g), with the advantage of being abundant and low-cost materials [22, 43, 130, 131]. Although aluminosilicates have a low SSA in comparison with activated carbons, sepiolite and bentonite have a good adsorption capacity for caffeine. For example, a sepiolite with a specific surface area of 221 m^2^/g has a maximum adsorption capacity of 48.7 mg/g [132], and bentonite with a low SSA (64.31 m^2^/g) has a *Q*_max_ of 42.5 mg/g [127]. The pH for caffeine adsorption in the subgroup of aluminosilicate mineral adsorbents (Table 9) varies between 6.0 and 6.6, values close to neutrality.

Soil and sediments (Table 10) are sandy and silty minerals obtained from aquifers and rivers. They are adsorbents of water contaminants, preferably those with a low content of organic matter and clay, which increase the specific surface area [50, 53, 136]. The maximum adsorption capacities of soils and sediments (in the range of 292 *μ*g/kg to 221.2 mg/g) tend to be lower than the *Q*_max_ of the other adsorbent types covered in this review. It has been also described that the equilibrium time among this group of adsorbents and adsorbates is very long, in some cases more than 24 h, which would explain why sediments do not retain ECs in rivers and water bodies [137, 138]. The pH for caffeine adsorption in this subgroup of inorganic adsorbents (Table 10) varies between 6.0 and 7.98, values close to neutrality.

#### 3.2.3. Composites

This subgroup comprises adsorbents formed by the combination of materials of two or more types, such as organic and inorganic, to improve their morphological characteristics, specific surface area, and adsorption capacity [60, 109]. They fall in two categories: organic-organic composites (Table 11) and organic-inorganic composites (Table 12). Composite adsorbents have been investigated with promising results for the removal of dyes [61, 62], heavy metals [64], and emerging contaminants [65, 66].

Organic-organic composites used for the adsorption of caffeine include chitosan-graphene, chitosan-reduced graphene oxide, and chitosan-waste coffee-grounds. The synthesis of composites notably improves the specific surface area of adsorbents and increases the caffeine adsorption capacity. For example, when chitosan was used as an individual adsorbent, a specific surface area of 3.6 m^2^/g and a maximum adsorption capacity of 0.0062 mg/g were obtained [121], while the combination with graphene yields 214 m^2^/g and 14.8 mg/g, respectively [140]. For the chitosan-reduced graphene oxide composite, the SSA is not reported, but the *Q*_max_ of caffeine was 63.6 mg/g [126]. The resin resulting from the copolymerization of N-vinyl-2-pyrrolidinone (PVC), ethylene glycol dimethacrylate (DEGMA), and triallyl isocyanurate (TAIC) has also been tested as a caffeine-adsorbent material, although with low removal capacity (2 mg/g) [141]. The pH range for caffeine adsorption on chitosan-graphene composites is 6-7, values close to neutrality.

Among organic-inorganic composites (Table 12), the best caffeine adsorbents found were MCM-48-GO, PSt/O-TiO_2_, and copper oxide nanoparticles on activated carbon [142–144]. The pH range for caffeine adsorption on the organic-inorganic composites was between 4 and 7, except for MgAl-LDH/biochar, with a pH of 12.

In general, in the group of organic adsorbents, activated carbons tend to have the highest specific surface area and *Q*_max_. Most of them are microporous in structure, although mesopores and macropores are also present, with a size distribution that mainly depends on three factors: origin of the raw material, type of activation, and duration of the activation process. The capacity of an activated carbon to retain a given substance depends not only depends on its specific surface area but also on the proportion of internal pores that the carbon has and the size of these pores (a suitable pore size should be between 1 and 5 times the diameter of the molecule to be retained) [150]. Adsorbents obtained from agricultural residues, biochar, and polymeric resins are modified by different processes developing porous structures that notably improve the maximum adsorption capacities, as it happens with the AC obtained from lignocellulosic materials [55]. Regarding composite-type adsorbents, Delhiraja et al. [140] have used functionalized graphene oxide composites (GO) for the adsorption of caffeine and other pharmaceutical and personal care products. Density functional theory calculations indicated that the adsorption mechanism is typically accompanied by size-related diffusion and a minor contribution of synergetic combination of hydrophobic/hydrophilic, hydrogen bonding, electrostatic, and *π*-*π* interactions.

## 4. Final Discussion

This section presents an analysis of the maximum adsorption capacity of caffeine on each adsorbent subgroup and its relationship with the specific surface area of the material and the pH at which adsorption was performed. The adsorption of caffeine-like molecules onto activated carbon, its interaction with adsorbents, and the regeneration and reuse of adsorbents for caffeine removal are also analyzed.

### 4.1. Maximum Adsorption Capacity

**Figure 5:**
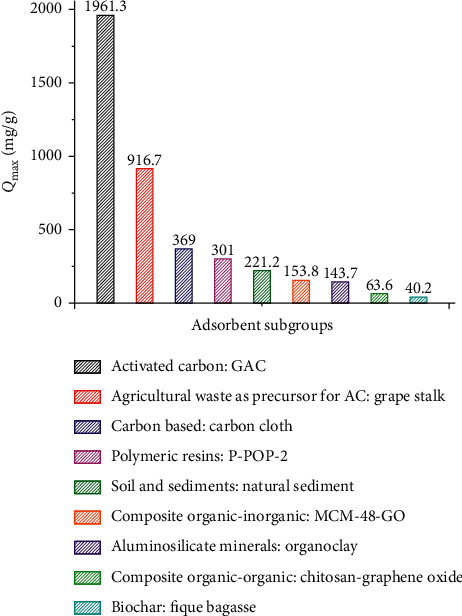
Maximum caffeine adsorption capacity per adsorbent subgroup.

Figure 5 shows the *Q*_max_ of caffeine for the adsorbents of each subgroup. Granular activated carbon (MG 1050 from ChiemiVall-Spain, SSA of 1100 m^2^/g and an average particle size of 1 mm) is the adsorbent with the highest adsorption capacity (1961.3 mg/g) followed by grape stalk activated carbon (916.7 mg/g) and activated carbon cloth (369 mg/g). The above three adsorbents are activated carbon-based organic adsorbents, and biochar is one of the organic adsorbents with the lowest *Q*_max_. Adsorbents such as natural sediment (221 mg/g) and aluminosilicate minerals (143.7 mg/g) are an option for removal of caffeine, due to their low cost and abundance.

**Figure 6:**
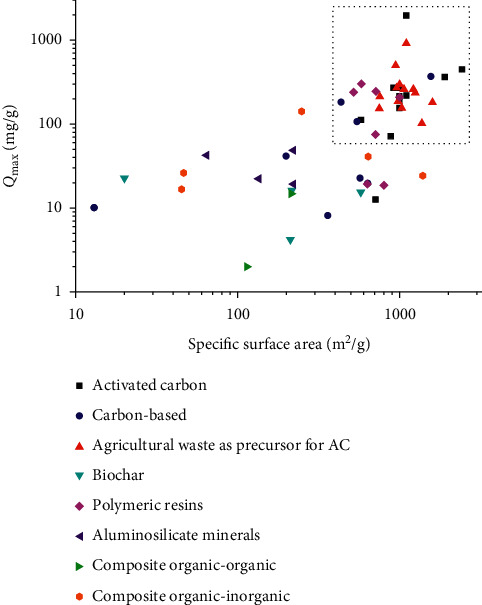
Relationship between the maximum adsorption capacity and specific surface area by adsorbent subgroups.

Figure 6 shows the *Q*_max_-SSA relation for the adsorbent subgroups. The highest *Q*_max_ values, above 70 mg/g, correspond to activated carbons (carbon-based and from agricultural wastes) followed by polymeric resins and organic-inorganic composites, with SSA in the 140–1900 m^2^/g range. A direct relationship between SSA and *Q*_max_ was found only for some adsorbents of the activated carbon subgroup.

**Figure 7:**
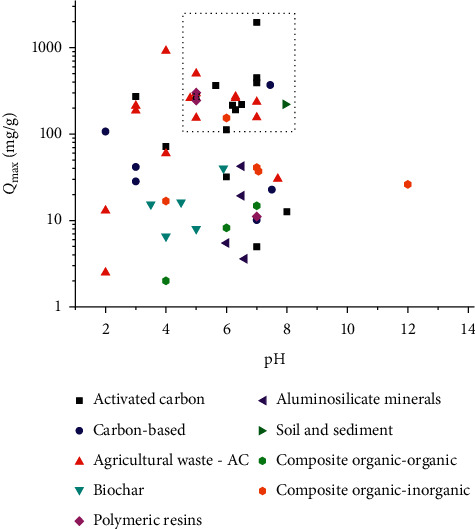
Relationship between the maximum adsorption capacity by adsorbent subgroups and pH.

In the publications covered in this review, the adsorption of caffeine has been performed over a wide pH range (2–10), although it is customary to operate at pH values close to neutral, and even some studies do not evaluate the influence of pH [47, 84, 151].  Figure 7 illustrates how the highest *Q*_max_ (activated carbons, included those produced from agricultural wastes) correspond to pH values between 5 and 7. It is also observed that the adsorption with carbon-based materials decreases with pH, and their higher *Q*_max_ are in the 2-3 pH range. It is important to consider that pH affects the degree of caffeine ionization and the surface charge of the adsorbent [99]. On some carbonaceous adsorbents, pH has little effect on caffeine adsorption; however, some studies indicate that caffeine adsorption capacity decreases with higher values of pH due to electrostatic repulsion [47, 48, 92, 133].

Regarding the adsorbent dose, its effect was not studied in most of the articles reviewed, but it was set in a very wide range, from 0.05 mg/L to 40000 mg/L, while the value used for the highest *Q*_max_ (1961.3 mg/L, with activated carbon) was 1000 mg/L [68].

For most of the organic adsorbent subgroups, the adsorption isotherms were fitted with the Langmuir model followed by the Freundlich, Sips, Toth, and Liu models. The Dubinin–Radushkevich and Redlich–Peterson models were also used for fitting the adsorption isotherms of caffeine in the subgroups of aluminosilicate minerals and organic-inorganic composites.

### 4.2. Adsorption of Caffeine-Like Molecules

Adsorption of similarly sized molecules can be an indicative of a suitable material for caffeine removal (similar to caffeine such as pharmaceuticals, stimulants, and personal use products), as molecular size and pore distribution influence the adsorption process, particularly on activated carbons [49]. For example, caffeine and diclofenac both have spherical equivalent diameters of 6.9 Å and 7.7 Å, respectively, and their adsorption in presence of powder carbonaceous materials can be attributed to their volumes being considerably smaller than the pore size of such materials [72]. Sotelo et al. [72] studied the removal of caffeine and diclofenac as emerging contaminants with three powder carbonaceous materials: activated carbon (AC, *S*_BET_ = 997.0 m^2^/g, *V*_micro_ = 0.260 cm^3^/g), multiwalled carbon nanotubes (MWNT, *S*_BET_ = 162.2 m^2^/g, *V*_micro_ = 0.016 cm^3^/g), and carbon nanofibers (CNF, *S*_BET_ = 199.1 m^2^/g, *V*_micro_ = 0.012 cm^3^/g). Carbonaceous materials were effective for the removal of emerging contaminants since the size of these compounds is considerably smaller than the pore size of AC, MWNT, or CNF. *Q*_max_ values for compounds in ultrapure water on AC, MWNT, and CNF were 271.0, 41.6, and 28.3 mg/g for caffeine and 329.0, 41.4, and 29.9 mg/g for diclofenac, respectively.

In a subsequent study, Sotelo et al. [49] investigated the adsorption of caffeine and diclofenac by granular activated carbon F-400 (*S*_BET_ = 997 m^2^/g and *V*_micro_ = 0.26 cm^3^/g). The molecular size of caffeine is 0.98 × 0.87 nm compared to that of diclofenac, 0.97 × 0.96 nm. In competitive adsorption, adsorption capacities for caffeine and diclofenac were 190.9 and 233.9 mg/g, respectively. Both adsorption capacities decreased compared to the single adsorption system by 32.1% for caffeine and 29.1% for diclofenac. Caffeine and diclofenac molecules accessed similarly sized pores and directly competed for the same adsorption sites. Higher values of octanol-water partition coefficient of a diclofenac molecule might be responsible for the stronger affinity of the adsorbent surface.

Mailler et al. [152] explored the removal of 15 micropollutants (including pharmaceutical, stimulant, and personal use compounds) from wastewater treatment plant discharges using 4 types of activated carbons with different micro- and mesoporosity ratios. The highest average removal of the pollutants investigated was 52%, which was achieved with PB 170® (*S*_BET_ = 957 m^2^/g, *V*_micro__+__meso_ = 0.5066 cm^3^/g), an activated carbon with the highest specific surface area and volume of micro-mesopores. The lowest average removal (26%) was obtained with PC 1000® (*S*_BET_ = 458 m^2^/g, *V*_micro__+__meso_ = 0.2435 cm^3^/g), which is the activated carbon with the lowest specific surface area and volume of micro-mesopores. For the other two activated carbons (WP 235® and W 35®) with similar pore volume (0.4841 and 0.4876 cm^3^/g), the average removal was 45%. In conclusion, the removal of micropollutants is associated with chemical characteristics of the compounds and textural properties of the adsorbent material, being favored by adsorbents with high specific surface area and micro and mesoporous volume.

Gil et al. [74] studied the removal of six emerging contaminants from aqueous solutions using a commercial granular activated carbon as an adsorbent (*S*_BET_ = 578 m^2^/g, *V*_totalpore_ = 0.564 cm^3^/g, *V*_micro_ = 0.206 cm^3^/g). The *Q*_max_ calculated for salicylic acid, caffeine, diclofenac, and ibuprofen from the isotherm fitting to the Langmuir model were 33, 88, 64, and 34 mg/g, respectively. In conclusion, organic molecules used in this study had a similar chemical structure. Therefore, the behavior during the adsorption by the activated carbon will also be very similar. Finally, Zhang et al. [153] evaluated the performance of powdered activated carbon (PAC, *S*_BET_ = 852.94 m^2^/g) for removing 28 types of antibiotics from water. Results of the PAC adsorption experiments showed that the percentage of removal of 28 selected antibiotics ranged from 96.5 to 99.9% and 86.8–99.6% in deionized water and surface water, respectively.

### 4.3. Interaction of Caffeine with Adsorbents

The porous structure of the adsorbent material, energy heterogeneity, and surface chemical properties (presence of functional groups) are the main factors influencing the adsorption equilibrium [154]. Caffeine has a high dipole moment, and its positive charge of the nitrogen atom electrostatically interacts with any negatively polarized functional group [17]. The pKa of caffeine is 10.4, and the protonated form of this molecule in aqueous solution exists when pH < pKa [41].

In carbonaceous adsorbents, such as activated carbon or graphene, the carbon surface has polar groups with hydrophilic behavior such as −NH, −OH, −O, and −COO. Therefore, the adsorption of caffeine can be attributed to dipole-dipole interactions, where *π*-electrons and 2-nitrophenol aromatic rings of caffeine interact with the *π*-aromatic electrons present on the adsorbent surface [17, 41, 84]. Beltrame et al. [54] explained caffeine adsorption as the result of *π*-*π* interactions and the formation of hydrogen bonds between caffeine heterocyclic rings and the aromatic rings of activated carbon (from pineapple plant) in a pH range of 2–7.

The removal of caffeine when the pH is lower than the zero charge point of the adsorbent (PZC) has been explained by hydrogen bonding between the adsorbent and adsorbate, as the surface of the material has a predominantly positive charge density and caffeine would not be electrostatically attracted to it [72].

The adsorption of caffeine with noncarbon adsorbents has been focused on low-cost materials such as sediments, polymer resins, and aluminosilicates, whose application is favored by the chemical properties of caffeine (high pKa and dipole moment). In these materials, the interaction of caffeine with the adsorbate is attributed to H-bonds, dipole-ion interactions, electrostatic interactions, and Van der Waals interactions [17, 40, 41, 155].

The adsorption of caffeine on inorganic surfaces of the natural sandy sediment occurs because caffeine is positively ionized at the experimental pH (7.94) and the sediment surface is negatively charged. Furthermore, the distribution coefficient (Kd) of caffeine on the inorganic surface (Kd = 17.58) is greater than that on organic matter (Kd = 0.28), confirming that the interactions of caffeine with inorganic surfaces control its adsorption on the sediment [41, 53].

In aluminosilicates, adsorption is facilitated in the interlayer space, even more so when these types of adsorbents are modified by expanding the interlayer space with quaternary ammonium sales [32, 43]. However, caffeine was also removed in thermally modified bentonite at temperatures above 400°C, i.e., when it has undergone a collapse, so that, in such case, the interaction of caffeine does not occur in the interlayer space but on the surface of the bentonite [130].

### 4.4. Regeneration and Reuse of Adsorbents

Although the adsorption capacity is an essential criterion in the selection of an adsorbent, its regeneration after use should be considered in a lifecycle analysis of the material, but only three publications out of the 133 selected included an analyzed reuse [68, 86, 97]. Similarly, in only one of the 45 manuscripts selected by Bachmann et al. [41] in their review on the removal of caffeine from aqueous media by adsorption, adsorbent regeneration tests were performed.

Batista et al. [97] used rapeseed activated carbons prepared by chemical activation with K_2_CO_3_ for caffeine adsorption. Temperatures of 400, 500, and 600°C under N_2_ flow were selected to carry out the regeneration assays. The thermal treatment at 400°C did not allow an effective regeneration of the activated carbon, and only 30% of the original adsorption capacity for caffeine was retained. For activated carbons regenerated at 500 or 600°C, an almost complete recovery of the caffeine adsorption capacity was observed (>95%), even after the second regeneration cycle. The N_2_ adsorption isotherms of the samples obtained after two exhaustion-regeneration cycles showed that at high regeneration temperatures (500 and 600°C), the volume of micropores available is higher. It is important to consider that activated carbons are regenerated ex situ by heating or steaming, which is a high energy-consuming process and can limit the reuse of the adsorbent [41, 94].

Multiwalled carbon nanotubes were used for the adsorption of diclofenac and caffeine. The adsorbent used was separated from the solution by filtration and treated with 0.1 M HCl for 2 h to desorb the organic retained compounds. The solid was then separated from the acid solution by filtration, washed with deionized water, and dried in an oven at 60°C for 48 h before being reused in the adsorption experiments. The reusability of this adsorbent was studied, and it was found to maintain its adsorption capacity after one cycle of reuse. However, data only referred to diclofenac [86].

Discarded granular activated carbon from a drinking water treatment plant was used for the adsorption of different pharmaceutical compounds, specifically caffeine, ibuprofen, and diazepam. The results were very promising since the reused carbon adsorbed caffeine (initial concentration of 1.23 mg/L, bed height of 10 cm, and weight of 13.51 g) and achieved a removal of about 40%. The reused activated carbon achieved a better performance for pharmaceutical compounds elimination as powder after grinding (at a concentration of 0.5 g/L) than as granular carbon in column. Caffeine was removed in percentage higher than 90%. Thus, future applications of used granular activated carbon, particularly in the framework of a circular economy, may be possible [68].

Abdel-Aziz et al. [156] synthesized bimetallic zero-valent iron/copper nanoparticles (FB-nZVFe/Cu), having used this material for caffeine adsorption. FB-nZVFe/Cu was used during five successive times for the adsorption of caffeine (5 mg/L). Between each adsorption cycle, FB-nZVFe/Cu was immediately collected from solution by centrifugation, washed with ethanol, and dried at 45 C, before being used for the next adsorption recycle. The caffeine removal was 82, 78, 83, 83, 70, and 69% in the 1st, 2nd, 3rd, 4th, and 5th cycle of use, respectively. Although a decrease in the removal efficiency was found with each reuse cycle, the FB-nZVFe/Cu material offered a high potential to be repeatedly used for caffeine removal without a considerable decrease in its removal efficiency. This article was not included for the analysis of adsorbents since its publication occurred after the date established in the search strategy.

## 5. Conclusions

The systematic literature review of publications on caffeine adsorption indicates that it is a recent and developing research topic because caffeine is becoming an emerging contaminant found in different types of water (surface, drinking, and wastewater). Caffeine is listed, along with nicotine, paraxanthine, and cotinine, as an anthropogenic marker of contamination, hence the importance of researching on methods for its removal. Of the total number of publications analyzed, 91.7% are research articles, 93.8% are written in English, and 67.1% are concentrated in four journals: Science of the Total Environment, Chemosphere, Environmental Science, and Chemical Engineering Journal.

Adsorption is one of the most frequently used methods for the removal of emerging contaminants, including caffeine. Powdered activated carbon (PAC) and granular activated carbon (PAC) are the mainly studied adsorbents for removal of caffeine. Their wide use is associated with their high specific surface area (up to 2431 m^2^/g) and high adsorption capacities (up to 1961.3 mg/g).

The high cost of activated carbon and its difficult regeneration have led to the search for low-cost, widely available, and ecofriendly adsorbents. Materials obtained from agricultural biomass waste, polymeric resins, clay minerals, soil and sediments, and organic-inorganic compounds emerge as an alternative adsorbent for the removal of caffeine. However, high doses (100 to 40000 mg/L) are required to achieve high removal of caffeine.

In 49% of the investigations analyzed in this review, the adsorption isotherms for caffeine fit into the Langmuir model, which assumes that adsorption takes place in a monolayer and that there is homogeneity on the surface of the adsorbent.

The adsorption capacity of caffeine depends on the properties of the adsorbent and other factors such as pH, adsorbate concentration, agitation speed, and contact time. Removal of caffeine increased with increasing adsorbent dose (or adsorbent amount) since the number of adsorption sites is greater. However, the capacity, in some cases, decreased with an increasing dose. This is because of the aggregation of particles, which leads to a decrease in active sites for adsorption. Determining the adsorbent dose is a useful factor in predicting the cost of the process per unit of solution to be treated.

## Figures and Tables

**Table 1:** Distribution of publications by type, area, and country.

Type	(%)	Area	(%)	Country	(%)
Article	91.7	Chemistry	25.7	China	23.3
Review	4.0	Environmental Science	17.3	Spain	17.5
Conference	2.8	Chemical Engineering	13.3	USA	12.0
Other^*∗*^	1.5	Biochemistry	11.1	Brazil	8.4
Book	0.0	Materials Science	7.5	France	5.5
Book chapter	0.0	Engineering	6.4	Other	33.3
		Other	18.7		

^*∗*^Data paper, book paper.

**Table 2:** Ranking of academic institutions by number of publications (NP).

Rank	Institution	Country	NP
1	Complutense University of Madrid	Spain	10
2	University of Lisbon	Portugal	8
3	Xi'an University of Architecture and Technology	China	6
4	University of South Carolina (USA)	USA	3
5	University of Cyprus	Cyprus	3
6	West Virginia University	USA	2

**Table 3:** Ranking of most cited research articles (excluding reviews).

Rank	Title	Number of citations	Year	Ref
1	Adsorption characteristics of selected hydrophilic and hydrophobic micropollutants in water using activated carbon	179	2014	[47]
2	Adsorption of pharmaceutical pollutants onto graphene nanoplatelets	129	2014	[48]
3	Competitive adsorption studies of caffeine and diclofenac aqueous solutions by activated carbon	115	2014	[49]
4	Potential for biodegradation and sorption of acetaminophen, caffeine, propranolol and acebutolol in lab-scale aqueous environments	107	2010	[50]
5	Removal of caffeine and diclofenac on activated carbon in fixed bed column	97	2012	[30]
6	Chemical-activated carbons from peach stones for the adsorption of emerging contaminants in aqueous solutions	87	2015	[51]
7	Synthesis of carbon xerogels and their application in adsorption studies of caffeine and diclofenac as emerging contaminants	75	2015	[52]
8	Sorption/desorption of non-hydrophobic and ionisable pharmaceutical and personal care products from reclaimed water onto/from a natural sediment	73	2013	[53]
9	Adsorption of caffeine on mesoporous activated carbon fibers prepared from pineapple plant leaves	67	2018	[54]
10	Activated carbons prepared from industrial pre-treated cork: Sustainable adsorbents for pharmaceutical compounds removal	64	2014	[55]

**Table 4:** Summary of characteristics of activated carbons and conditions of the adsorption process.

Type	SSA (m^2^/g)	Dose (mg/L)	pH	*Q*_max_ (mg/g)	IM	Ref
GAC	1100	1000	7	1961.3	Langmuir	[68]
Hydrothermal carbons	2431	200	7	448.4	Langmuir	[69]
F-400	—	60	7	393.7	Langmuir	[70]
AC from polymer waste	1900	100	5.65	363.6	Langmuir	[67]
GAC	917	6.7	5	271.1	Langmuir	[71]
PAC	997	—	3	271.0	Langmuir	[72]
F-400	1102	—	6.5	219.2	—	[33]
F-400	997	800	6.2	214.7	—	[73]
F-400	997	—	6.3	190.9	Freundlich	[49]
GAC	997	800	—	155.6	Freundlich	[30]
GAC	578	50	6	112	Toth	[74]
PAC	882.6	10	4	71.7	Freundlich	[75]
GAC	—	10000	6	31.94	Langmuir	[76]
PAC	710.4	20	8	12.63	Langmuir	[77]
PAC	—	500	7	4.95	—	[78]
GAC	—	900	7.9	396 *μ*g/g	Langmuir	[79]
PAC	1256	540	—	27 *μ*g/g	Freundlich	[80]
GAC	1000	3000	7.3	161 ng/g	—	[81]

**Table 5:** Summary of characteristics of carbon-based adsorbents and conditions of the adsorption process.

Type	SSA (m^2^/g)	Dose (mg/L)	pH	*Q*_max_ (mg/g)	IM	Ref
Carbon cloth	1560	12	7.45	369.0 (1.9 mmol/g)	Langmuir	[85]
Carbon xerogels treated with urea solution	435	60	—	182.5	Sips	[52]
Carbon xerogel modified with (CH_3_COO)_2_Cu	546	20	2	107.0	Langmuir	[87]
Carbon nanotubes	199.1	—	3	41.6	Langmuir	[72]
Carbon nanofibers	162.2	—	3	28.3	Langmuir	[72]
Graphene	570.2	100	7.5	22.7	Langmuir	[84]
Graphene nanoplatelets	635.2	200	—	19.72	—	[48]
Commercial column C_18_	—	200	—	11.35	Freundlich	[82]
Carbon nanotubes	13	50	7	10.1	Toth	[86]
Carbon nanotubes	360	—	—	8.14	—	[83]

**Table 6:** Summary of characteristics of agricultural wastes, directly as adsorbents or as precursors for activated carbon, and conditions of the adsorption process.

Source	SSA (m^2^/g)	Dose (mg/L)	pH	*Q*_max_ (mg/g)	IM	Ref
Grape stalk-AC	1099.86	—	4	916.7	Sips	[92]
Pine activated-AC	945	6	5	500	Langmuir	[91]
Biodiesel production waste-AC	1165	6	5	296.3	Langmuir	[97]
Peach stones modified by oxidation-AC	959	120	6.3	270.0	Sips	[51]
Peach stones-AC	1216	120	6.3	260.0	Sips	[51]
*Eragrostis plana* (Nees leaves)-AC	1250	70	7	235.5	Liu	[95]
Dende coco-AC	755	10	3	212.3	Langmuir	[94]
Babassu coco-AC	980	10	3	186.9	Langmuir	[94]
Biomass impregnated KOH-AC	1597	10	—	181.23	Freundlich	[98]
Pineapple plant leaves-AC	1031	25	7	155.5	Langmuir	[54]
Industrial pretreated cork-AC	750	6	5	153.4	—	[55]
Peach stones under helium-AC	1064	120	4.8	260	Sips	[51]
Biomass-AC	1373	10	—	102.04	Langmuir	[98]
*Luffa cylindrica*-AC	—	50	4	59.9	Langmuir	[99]
*Acacia mangium* wood-AC	—	3000	7.7	30.3	—	[96]
*Elaeis guineensis*-AC	407.66	200	2	13	Langmuir	[93]
Date stone (*Phoenix dactylifera*)-AC	—	8000	—	8.7	—	[100]
*Eichhornia crassipes*-water hyacinth	—	1200	2	2.49	Langmuir	[101]

**Table 7:** Summary of characteristics of biochar adsorbents and conditions of the adsorption process.

Biochar base	SSA (m^2^/g)	Dose (mg/L)	pH	*Q*_max_ (mg/g)	IM	Ref
Fique bagasse	211.7	10000	5.9	40.2	—	[107]
Pistachio shells	20	10	—	22.6	Langmuir	[108]
*Gliricidia sepium*	216.4	1000	4.5	16.26	Freundlich	[102]
Tea-waste	576	1	3.5	15.4	Freundlich	[109]
Rice husk	144	50	5	8	Langmuir	[110]
Pine needles	—	50	4	6.54	Langmuir	[111]

**Table 8:** Summary of characteristics of polymeric resins and biopolymers and conditions of the adsorption process.

Adsorbent	SSA (m^2^/g)	Dose (mg/L)	pH	*Q*_max_ (mg/g)	IM	Ref
*Polymeric resin*						
P-POP-2	581	200	5	301	Langmuir	[60]
P-POP-1	714	200	5	245	Langmuir	[60]
GS18 (MAR)	480–520	33333	—	239.9	Freundlich	[112]
XDA-200	1000	40000	—	209.0	Freundlich	[113]
D101	710.1	1000	—	75.2	Langmuir	[114]
Amberlite® XAD-7	450	—	7	58.32	Langmuir	[115]
MIP	—	20000	—	39.65	—	[82]
Copolymer divinylbenzene-acrylonitrile	632	4800	—	19.3	Freundlich	[116]
NIPAAm-based hydrogels	—	—	—	19 mg/mL	Langmuir	[44]
Oasis® HLB	800	200	—	18.64 (96 mmol/kg)	Langmuir	[117]
Polyvinylpolypyrrolidone	—	500	7	11.09	Langmuir	[118]
Macroporous crosslinked polyvinyl alcohol	700–800	500	—	7.73	—	[119]
Resinex/SR 5500	861	5.4 mL/L	7	256.4 ng/g	Langmuir	[120]

*Biopolymer*						
Chitosan	3.6	1000	7	0.00617	Langmuir	[121]

**Table 9:** Summary of characteristics of aluminosilicate mineral adsorbents and conditions of the adsorption process.

Adsorbent	SSA (m^2^/g)	Dose (mg/L)	pH	*Q*_max_ (mg/g)	IM	Ref
Organoclay (montmorillonite)	—	50	—	143.7	Langmuir	[43]
Organoclay (sepiolite)	—	50	—	134.0 (0.69 mmol/g)	Langmuir	[43]
Montmorillonite	—	—	—	122.4 (0.63 mmol/g)	Langmuir	[22]
Na-montmorillonite	—	400	—	120.4 (0.62 mmol/g)	Langmuir	[131]
Montmorillonite (calcinated at 200°C)	—	4000	—	99.0 (0.51 mmol/g)	Langmuir	[130]
Sepiolite	221	1600	—	48.7	Langmuir	[132]
Bentonite	64.31	—	6.5	42.5	—	[127]
Bentonite	135	100	—	22.3	Langmuir	[59]
Sepiolite	—	2500	—	20	Dubinin-Radushkevich	[133]
Sepiolite	221	—	6.5	19.27	—	[33]
Clinoptilolite		100	6	5.48	Langmuir	[134]
Bentonite	—	2500	6.6	3.6	Freundlich	[42]
Calcined Verde-lodo	—	500	—	8.78 *μ*mol/g	—	[135]
Spectrogel	—	500	—	3.27 *μ*mol/g	—	[135]
Fluidgel organoclay	—	500	—	2.12 *μ*mol/g	—	[135]
Calcined fluidgel		500	—	2.73 *μ*mol/g	—	[135]

**Table 10:** Summary of characteristics of soil and sediment adsorbents and conditions of the adsorption process.

Adsorbent	SSA (m^2^/g)	Dose (mg/L)	pH	*Q*_max_ (mg/g)	IM	Ref
Natural sediment	—	50	7.94	221.2	Freundlich	[53]
Subsoil	—	31.5	6	7.2 *μ*g/g	—	[138]
Sediment	15.21	10	7.5	444 *μ*g/kg	Langmuir	[136]
Sediment	6.1	100	7	360 *μ*g/kg	Freundlich	[50]
Natural soil	—	300	7.98	292 *μ*g/kg	—	[137]
Sediment		15	7.50	—	Freundlich	[139]

**Table 11:** Summary of characteristics of the composite adsorbent organic-organic types and conditions of the adsorption process.

Adsorbent	SSA (m^2^/g)	Dose (mg/L)	pH	*Q*_max_ (mg/g)	IM	Ref
Chitosan-reduced graphene oxide	—	3750	—	63.6	—	[126]
Graphene-chitosan	214	25	7	14.8	Langmuir	[140]
Chitosan/waste coffee-grounds	—	50	6	8.21	Freundlich	[123]
PVP–DEGMA–TAIC	114	0.05	4	2	Langmuir	[141]

**Table 12:** Summary of characteristics of composite adsorbent organic-inorganic types and conditions of the adsorption process.

Adsorbent	SSA (m^2^/g)	Dose (mg/L)	pH	*Q*_max_ (mg/g)	IM	Ref
MCM-48-GO	—	40	6	153.8	Langmuir	[142]
PSt/O-TiO_2_	248.5	1000	—	141.69	Freundlich	[143]
Copper oxide nanoparticles on activated carbon	640	100	7	41.0	—	[144]
Lignocellulosic residues impregnated with TiO_2_	—	7000	7.06	37.1	Langmuir	[145]
MgAl-LDH/biochar	46.43	4000	12	26.2	Redlich–Peterson	[146]
UiO-66	1391	3	—	24.25	—	[147]
Polypyrrole-Fe_3_O_4_@SiO_2_	45.08	60	4	16.74	Langmuir-Freundlich	[148]
Cu^2+^ amino grafted SBA-15 mesoporous silica	—	15	7	0.25 *μ*g/g	Freundlich	[149]

## Data Availability

All data used to support the findings of this study are included within the article.

## Abbreviations

AC: Activated carbon
C18: Column that uses octadecylsilane as the stationary phase
CFN: Carbon nanofibers
D101: Macroporous resin of nonpolar styrene-co-divinylbenzene copolymer groups
DEGMA: Ethylene glycol dimethacrylate
ECs: Emerging contaminants
F-400: Granular activated carbon (Filtrasorb 400)
FB-nZVFe/Cu: Bimetallic zero-valent iron/copper nanoparticles
GAC: Granular activated carbon
GO: Graphene oxide
GS18: Styrene-divinyl benzene
IM: Isotherm model
Kd: Distribution coefficient
Kow: Octanol-water partition coefficient
LDH: Layered double hydroxide
MAR: Macroporous adsorption resin
MCM-48: Class of ordered mesoporous silica with cubic symmetry
MG 1050: Granular activated carbon (mesoporous) from Chiemivall-Spain
MIP: Molecular imprinted polymer
MWNT: Multiwalled carbon nanotubes
NIPAAm: N-isopropylacrylamide
Oasis® HLB: Universal polymeric reversed-phase sorbent cartridge (water corporation)
PAC: Powdered activated carbon
PB 170®: Activated carbon commercialized by DaCarb® (France)
PC 1000®: Activated carbon commercialized by DaCarb® (France)
pKa: −logKa, Ka: acid dissociation constant
P-POP: Phosphate-based porous organic polymers
P-POP-1: P-POP, diphenyl phosphate and 1,1,2,2-tetraphenylethylene as precursor
P-POP-2: P-POP, diphenyl phosphate and 1,3,5-triphenylbenzene as precursors
PSt: Polystyrene microbeads
PVP: N-vinyl-2-pyrrolidinone
PZC: Point zero charge
*Q*_max_: Maximum adsorption capacity
SBA-15: Class of ordered mesoporous silica, Santa Barbara Amorphous-15
SR 5500: Epoxy resin 5500 (Sicomin Epoxy Systems)
*S*_BET_: Specific BET surface, BET (Brunauer, Emmett, and Teller)
SSA: Specific surface area
TAIC: Triallyl isocyanurate
UiO-66: Zirconium-based MOF
WP 235®: Activated carbon commercialized by Chemviron® (Belgium)
W 35®: Activated carbon commercialized by Norit® (Netherlands)
XAD: Macroporous resin with a styrene-divinyl benzene structure and hydroxyl functional groups
XAD-7: Amberlite, matrix: aliphatic carboxylic acid polymer
XDA-200: Hyper-crosslinked polystyrene macroporous.
